# Conventional and Pro-Inflammatory Pathways of Fibrinolytic Activation in Non-Traumatic Hyperfibrinolysis

**DOI:** 10.3390/jcm11247305

**Published:** 2022-12-09

**Authors:** Johannes Zipperle, Bernhard Ziegler, Herbert Schöchl, Wolfgang Voelckel, Peter Dungel, Janne Cadamuro, Marcin Osuchowski, Christoph J. Schlimp, Daniel Oberladstätter

**Affiliations:** 1 Ludwig Boltzmann Institute for Traumatology, The Research Center in Cooperation with AUVA, 1200 Vienna, Austria; 2 Department of Anaesthesiology, Perioperative Medicine and General Intensive Care Medicine, Paracelsus Medical University, 5020 Salzburg, Austria; 3 AUVA Trauma Centre Salzburg, Department of Anaesthesiology and Intensive Care Medicine, Academic Teaching Hospital of the Paracelsus Medical University, 5020 Salzburg, Austria; 4 Department of Laboratory Medicine, University Hospital SALK, 5020 Salzburg, Austria; 5 AUVA Trauma Centre Linz, Department of Anaesthesiology and Intensive Care Medicine, 4010 Linz, Austria

**Keywords:** thromboelastometry, viscoelastic tests, cardiac arrest, hyperfibrinolysis, inflammation, activated protein C, neutrophil extracellular traps, ischemia, reperfusion

## Abstract

Hyperfibrinolysis (HF) frequently occurs after severe systemic hypoperfusion during major trauma and out-of-hospital cardiac arrest (OHCA). In trauma-induced HF, hypoperfusion, the activation of protein C (APC), and the release of tissue plasminogen activator (t-PA) have been identified as the driving elements of premature clot breakdown. The APC pathway also plays a role in inflammatory responses such as neutrophil extracellular trap formation (NETosis), which might contribute to lysis through cleavage of fibrin by neutrophil elastases. We investigated whether the APC and the plasminogen pathway were general drivers of HF, even in the absence of a traumatic incident. Additionally, we were interested in inflammatory activation such as the presence of NETs as potential contributing factors to HF. A total of 41 patients with OHCA were assigned to a HF and a non-HF group based on maximum lysis (ML) in thromboelastometry. Thrombin–antithrombin (TAT)-complex, soluble thrombomodulin (sTM), APC–PC inhibitor complex, t-PA, PAI-1, t-PA–PAI-1 complex, plasmin–antiplasmin (PAP), d-dimers, neutrophil elastase, histonylated DNA (hDNA) fragments, and interleukin-6 were assessed via immunoassays in the HF group vs. non-HF. APC–PC inhibitor complex is significantly higher in HF patients. Antigen levels of t-PA and PAI-1 do not differ between groups. However, t-PA activity is significantly higher and t-PA–PAI-1 complex significantly lower in the HF group. Consistent with these results, PAP and d-dimers are significantly elevated in HF. HDNA fragments and neutrophil elastase are not elevated in HF patients, but show a high level of correlation, suggesting NETosis occurs in OHCA as part of inflammatory activation and cellular decay. Just as in trauma, hypoperfusion, the activation of protein C, and the initiation of the plasminogen pathway of fibrinolysis manifest themselves in the HF of cardiac arrest. Despite features of NETosis being detectable in OHCA patients, early pro-inflammatory responses do not appear be associated with HF in cardiac arrest.

## 1. Introduction

Hyperfibrinolysis (HF) occurs after both severe injury and out-of-hospital cardiac arrest (OHCA), suggesting hypoperfusion as the common denominator of fibrinolytic activation [1,2]. Trauma, ischemia, and reperfusion trigger a vast array of responses at the interface between hemostasis and inflammation [3]. Upon injury, blood loss can be aggravated by trauma-induced coagulopathy (TIC), a multi-factorial dysregulation of hemostasis that results in a clinical bleeding tendency [4]. TIC is considered a consequence of tissue trauma, hypoperfusion, and the depletion of functional coagulation factors and platelets [5]. Tissue trauma itself results in the release of damage-associated molecular patterns (DAMPS) and subsequent inflammatory responses such as endothelial activation. These conditions affect blood coagulation by altering endothelial barrier function and the expression of cell-surface molecules [6,7,8]. Another crucial confounder is the activation of fibrinolytic pathways that are capable of degrading the newly formed fibrin clot at the site of injury. The manifestation of fibrinolytic activation in hyperfibrinolysis (HF) is considered a severe complication after trauma, which is independently associated with a poor outcome [9]. Fibrin degradation is primarily orchestrated by plasmin, which is activated from plasminogen by tissue plasminogen activator (t-PA). T-PA is stored in endothelial Weibel–Palade bodies and is released into the circulation in response to stimuli such as hypoxia, bradykinin, and thrombin [10]. A major driver of the HF of trauma is protein C, a serine protease with essential functions in endogenous anticoagulation, inflammation, and cytoprotection [11,12]. Circulating protein C exists as zymogen; it is activated by the endothelial protein C receptor (EPCR) together with thrombomodulin (TM) at the inner surface of blood vessels, where it also functions as an endogenous anticoagulant. Activated protein C (APC) is capable of modulating fibrinolysis by binding to PAI-1, by enhancing t-PA function, and by cleaving the coagulation factors Va and VIIIa [13]. Apart from this conventional regulation of fibrinolysis, APC also fulfills inflammatory functions as it prevents neutrophils from undergoing cell death programs such as NETosis [14]. NETosis is a specialized, lethal process in neutrophils that results in the release of a network of DNA, histones, elastases, and myeloperoxidases and aims to entangle and immobilize pathogenic invaders [15]. Neutrophils not only play a key role in pathogen clearance but also thrombus formation and breakdown at the fluent boundaries between coagulation, inflammation, and tissue remodeling [16]. Aside from trauma, HF generally appears to occur in conditions that are associated with systemic ischemia [17]. Hypo- and reperfusion also result in the priming and recirculation of activated neutrophils that become sequestrated in bystander organs and the microcirculation [18]. Neutrophils possess a vast array of proteolytic enzymes, such as elastases, known to be capable of degrading fibrin and other fibrillar matrix proteins [19]. The activation of the PC pathway, together with fibrinolytic activation and the precursors of a profound pro-inflammatory response, might drive fibrinolysis towards conventional and inflammatory downstream targets, even in the absence of a traumatic injury. In this study, we hypothesized that the APC pathway, together with conventional fibrinolytic mediators, drives HF also in the absence of tissue trauma, and that neutrophil serine proteases contribute to this mechanism in a state of severe systemic hypoperfusion.

## 2. Materials and Methods

### 2.1. Study Design

The study was approved by the local ethics committee of the state of Salzburg (Votum Nr. E 1143-09) and was carried out between January 2010 and April 2011. Patient samples were collected as part of the prospective hyperfibrinolysis in cardiac arrest (HICA) study that investigated the incidence of hyperfibrinolysis in out-of-hospital cardiac arrest (OHCA) patients. A detailed overview of patient demographics and clinical metrics is published elsewhere [2]. Informed consent was waived by the authorized ethics committee. Patients were enrolled when spontaneous breathing and a palpable pulse were absent, independent of the duration between collapse and the initiation of life support measures. Blood was drawn on scene by an emergency physician after OHCA had been determined and CPR was started. Patients were excluded when one of the following criteria were applicable: age under 18, cardiac arrest caused by trauma, pregnancy, and participation in another clinical study. For thromboelastometry (ROTEM) and some ELISA measurements, 3 mL of blood was collected in tubes containing 0.3 mL buffered 3.2% trisodium citrate, resulting in a volume ratio of 1:10 (S-Monovette^®^, Sarstedt, Nümbrecht, Germany). For ELISA kits that required EDTA as an anticoagulant, plasma was also obtained from blood that was collected in tubes with 1.6 mg mL-1K3-EDTA (S-Monovette^®^, Sarstedt, Nümbrecht, Germany). All samples were processed within 2 h.

### 2.2. Viscoelastic Testing

Data on the incidence and extent of fibrinolytic activation were retrieved from the HICA study’s central database. In the HICA study, a tissue-factor-activated thromboelastometry (exTEM^®^) was performed with an automated pipet according to the manufacturer’s instructions. The exTEM^®^ assay uses tissue factor to not only fully activate plasmatic coagulation via factor VII, but also to maximize fibrinolytic activation in the sample. ROTEM delta (TEM Innovations, Munich, Germany) is capable of determining the changes in viscoelastic properties as a blood clot forms in the measurement cup of the device. Initiation and dynamics of clot formation, as well as a potential breakdown of the clot, can be derived from the recorded tracings. Measurements were performed for at least 60 min with an emphasis on maximum lysis (ML, defined as the relative decrease in clot strength after the maximum clot firmness (MCF) had been generated, physiological range 5–15%), a primary indicator of fibrinolytic activation. Hyperfibrinolysis was diagnosed when ML exceeded 15%.

### 2.3. Detection of Mediators by Immunoassays

Plasma was generated from patient blood samples by centrifugation (2500× *g* for 15 min) and was frozen at −80 °C immediately. Enzyme-linked immunosorbent assays (ELISAs) were performed according to respective instruction manuals to determine mediators of hemostasis and inflammation. Tissue plasminogen activator (t-PA) Combi Actibind, as well as plasminogen activator inhibitor-1 (PAI-1) and t-PA–PAI-1 complex, ELISA kits were purchased from Technoclone (Vienna, Austria). An ELISA kit from USCN (USCN Life Sciences Inc., Wuhan, China) was used to determine plasmin–antiplasmin (PAP) complex levels in plasma. An activated protein C–protein C inhibitor (APC–PCI) complex kit was obtained from BioPorto diagnostics (Hellerup, Denmark) and soluble thrombomodulin was measured with a kit from Asserachrom (Diagnostica Stago, Asnieres, France). Interleukin-6 levels were measured with an Immulite 1000 device (Siemens Healthcare, Erlangen, Germany). Thrombin–antithrombin (TAT) complex was determined with the Enzygnost© TAT Micro-kit from Siemens (Siemens Healthcare, Erlangen, Germany). Furthermore, ELISA measurements of histonylated, citrullinated DNA fragments (Cell Death Detection ELISA kit-plus, Roche, Basel, Switzerland) and PMN Elastase (alpha 1PI-complex, Milenia Biotec, Nauheim, Germany) were performed.

### 2.4. Statistical Analysis

Data were analyzed and plotted in Prism 5 (GraphPad Software Inc., La Jolla, CA, USA). All data from OHCA patients were stratified by ROTEM maximum lysis (ML < 15 vs. ML > 15) and analyzed for Gaussian distribution by a Kolmogorov–Smirnov test. Parameters from both groups were compared by Student’s *t*-test or a Mann–Whitney test with Welch correction whenever applicable. Patient data were displayed in Tukey box and whisker blots. The association of various parameters was analyzed by Spearman or Pearson correlation or regression, and expressed with *p* values rho or r2 where applicable. In the entire study, *p* values below 0.05 were considered significant.

## 3. Results

### 3.1. A Subgroup of OHCA Patients Present with Hyperfibrinolysis

A total of 53 patients were enrolled in the hyperfibrinolysis in out-of-hospital cardiac arrest (HICA) study and were eligible for this post-hoc analysis. Of these 53 enrolled patients, 41 patients (30 male, 11 female) had sufficient plasma volume remaining to perform all ELISA measurements. Based on the ROTEM tracings from the HICA dataset, 15 patients (of the 41) presented with non-traumatic HF (ML > 15%) while 26 patients displayed a maximum lysis < 15% (ML < 15%) and were, hence, assigned to the respective subgroups. An overview of basic patient characteristics as well as the performed immuno-assays is given in Table 1. Results from viscoelastic measurements in the HICA cohort were published in detail in the original study [2].

### 3.2. Non-Traumatic HF Is Associated with an Activation of the Protein C Pathway

**Figure 1:**
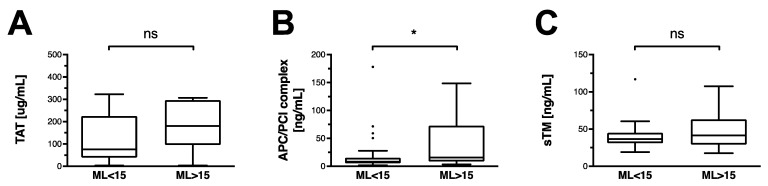
Thrombin–antithrombin, thrombomodulin, and activated protein C in OHCA patients. Data are shown as box and whiskers blots with Tukey-style depiction of outliers. OHCA patients were stratified by ROTEM maximum lysis (ML). Hyperfibrinolysis was defined as a ML > 15%. Parameters in patients with and without hyperfibrinolysis were compared with a Student’s *t*-test or Mann–Whitney test, where applicable. Comparison between (**A**) thrombin–antithrombin (TAT), (**B**) soluble thrombomodulin (sTM), and (**C**) activated protein C–protein C inhibitor (APC/PCI) complex levels in OHCA patients with and without hyperfibrinolysis. * = *p* < 0.05; ns = non-significant.

Thrombin formation, which is detected in a complexed form with antithrombin (TAT), is equal in the non-HF and the HF group (Figure 1A). In a similar fashion, soluble thrombomodulin levels are equal in both groups (Figure 1B). APC–PCI complex, a stable indicator for the activation of the protein C pathway, is significantly higher in HF patients (Figure 1B).

### 3.3. Conventional Fibrinolytic Pathways Are Activated in Non-Traumatic Hyperfibrinolysis

**Figure 2:**
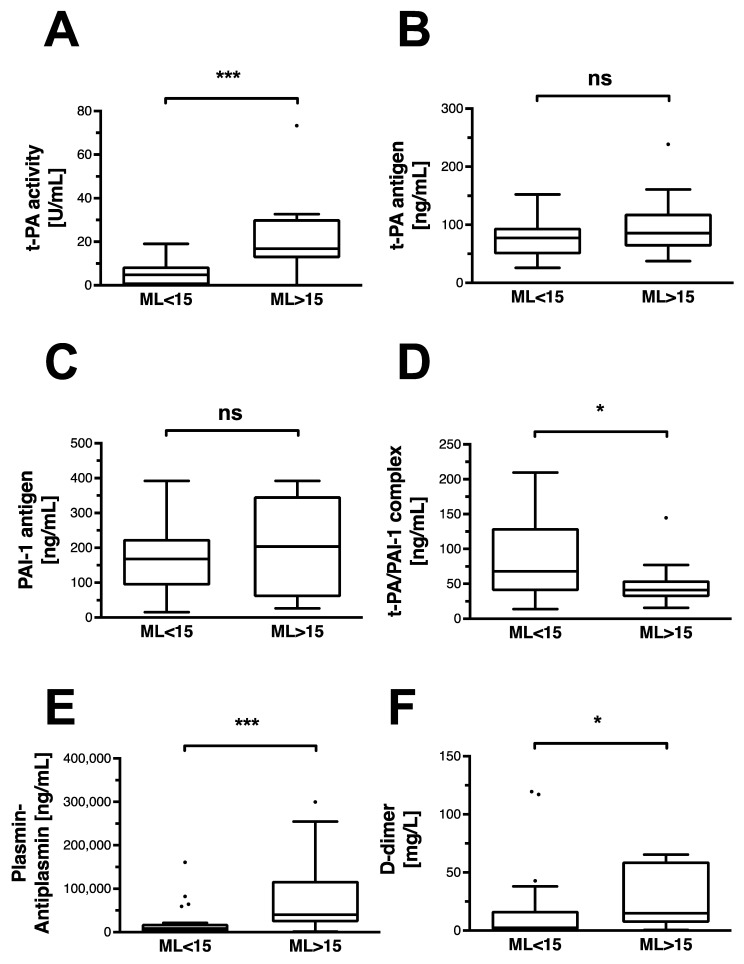
Classic fibrinolytic mediators and indicators of fibrinolysis in OHCA patients. Data are shown as box and whiskers blots with Tukey-style depiction of outliers. OHCA patients were stratified by ROTEM maximum lysis (ML). Hyperfibrinolysis was defined as a ML > 15%. Parameters in patients with and without hyperfibrinolysis were compared with a Student’s *t*-test or Mann–Whitney test, where applicable. Differences in (**A**) t-PA activity and (**B**) t-PA antigen levels in OHCA patients with and without hyperfibrinolysis. Comparison of (**C**) PAI-1 antigen and (**D**) its complex with t-PA in OHCA patients with and without hyperfibrinolysis. Difference in circulating (**E**) plasmin–antiplasmin (PAP) and (**F**) D-dimer levels in OHCA patients with and without hyperfibrinolysis. T-PA: tissue plasminogen activator; PAI-1: plasminogen activator inhibitor-1 * = *p* < 0.05; *** *p* < 0.001; ns = non-significant.

Activity of the circulating t-PA is significantly higher in the HF group (Figure 2A). In contrast, detectable antigen levels of t-PA are similar in the HF and in non-HF groups (Figure 2B). Similarly, PAI-1 antigen is not detectable at higher concentrations in any of the groups (Figure 2C). However, formation of its complex with t-PA is observable to a significantly lesser extent in the HF group (Figure 2D). The resulting activation of plasmin is detected through its complexed form with antiplasmin, and is significantly higher in HF patients (Figure 2E). D-dimers, as indicators of fibrin degradation, are detected at significantly higher levels in the HF group (Figure 2F).

### 3.4. Pro-Inflammatory Pathways Do Not Appear to Be Associated with Non-Traumatic HF 

**Figure 3:**
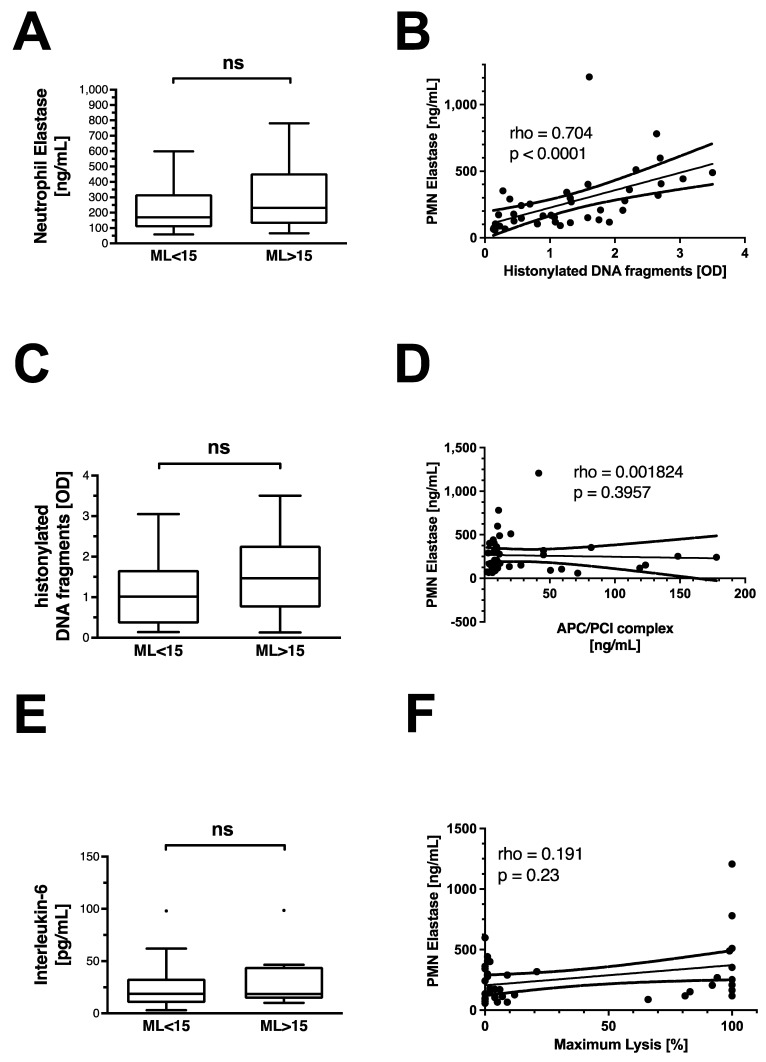
Inflammatory markers and features of neutrophil extracellular trap release in OHCA patients. Differences in (**A**) neutrophil elastase, (**C**) histonylated DNA fragments, and (**E**) interleukin-6 levels in OHCA patients with and without hyperfibrinolysis. Data are shown as box and whiskers blots with Tukey-style depiction of outliers. OHCA patients were stratified by ROTEM maximum lysis (ML). Hyperfibrinolysis was defined as a ML > 15%. Parameters in patients with and without hyperfibrinolysis were compared with a Student’s *t*-test or Mann–Whitney test, where applicable. Spearman correlation of (**B**) histonylated DNA fragments and neutrophil Elastase; (**D**) activated protein C–protein C inhibitor (APC/PCI) complex and neutrophil elastase; and (**F**) neutrophil elastase and ROTEM maximum lysis in all OHCA patients. PMN = polymorphonuclear neutrophil; OD = optical density; ns = non-significant.

Non-traumatic HF is not associated with an increase in circulating neutrophil elastase, histonylated DNA (hDNA) fragments, or interleukin-6 (Figure 3A,C,E). There is a strong correlation between the release of the NET components hDNA fragments and neutrophil elastase (Figure 3B). No correlation is observed between the APC–PCI complex and neutrophil elastase (Figure 3D), or neutrophil elastase with maximum lysis (Figure 3F). 

## 4. Discussion

In the current study, we demonstrate the role of conventional and inflammatory pathways of fibrinolytic activation in non-traumatic HF. Our findings indicate that profound tissue hypoperfusion is the common denominator in the pathogenesis of both traumatic and non-traumatic HF, also with regard to pathophysiological mechanisms. APC is significantly elevated in HF, which is accompanied by the release of t-PA. In HF patients, the available t-PA elicits higher activity, as it is complexed with PAI-1 to a lesser extent in HF. Consequently, the more prominent activation of plasminogen by t-PA is demonstrated by higher levels of d-dimers and plasmin–antiplasmin concentration in the plasma of HF patients. There is no evidence for an association of HF with early pro-inflammatory responses such as IL-6 release or NETosis. Despite a lack of association with HF, histone-complexed DNA strongly correlates with the presence of neutrophil elastase in plasma, suggesting NET formation occurs in OHCA. Since NETs are usually deposited on surfaces, granular enzymes that are immobilized within NETs may, therefore, not necessarily be detectable in the plasmatic phase of a blood sample. Nevertheless, we demonstrate a strong correlation of histone-complexed DNA with neutrophil elastase in plasma from OHCA patients.

Cardiac arrest represents a life-threatening condition that is accompanied by systemic ischemia and cellular decay. Aside from that, it is also associated with a severe derailment of hemostasis, the latter comprising numerous anticoagulant and fibrinolytic pathways. In cardiac arrest patients with HF, we observe elevated levels of the APC/PCI complex. APC/PCI was chosen as an antigen in immunoassays due to a longer half-life of APC as a complex with its dedicated inhibitor. In its membrane-bound form, TM serves as a crucial co-factor in the activation of protein C. Interestingly, although it also represents a well-established marker of endotheliopathy in the critically ill, it is not significantly elevated in HF [20,21]. TM’s downstream target APC is a known driver of HF in trauma, and has been reported to prevent neutrophils from undergoing NETosis. Our findings suggest that APC also plays a role in HF in the absence of a traumatic event, substantiating the importance of hypoperfusion in the pathogenesis of HF [22].

However, we do not establish a connection between APC’s function as an endogenous anticoagulant and its cytoprotective role in leukocytes. Nevertheless, we also report a strong correlation between histonylated DNA fragments and neutrophil elastase, the main components of neutrophil extracellular traps. Since there is no correlation between APC–PCI levels and neutrophil elastase, we suggest APC’s anticoagulant role as more prominent in non-traumatic HF. Major trauma typically results in a release of cellular damage markers and a rapid inflammatory response characterized by the release of pro/anti-inflammatory cytokines. However, in the absence of a traumatic cause of OHCA, this early pro-inflammatory mechanism does not appear to be associated with the manifestation of HF. Due to immune cell dysregulation and ischemia–reperfusion injury, severely traumatized patients often also display an established leukocytosis with neutrophilia [23,24]. Activated neutrophils are capable of evading the circulation to tissues exposing a rich armamentarium of proteolytic enzymes for degradation of matrix proteins and phagocytosis. These mechanisms have long been known to contribute to fibrin breakdown through direct degradation of the fibrin mesh on one side and the cleavage of fibrinolytic pro-enzymes on the other [25]. Some of these serine proteases are released during NETosis, along with histone-complexed DNA [26]. These complexes can also be found in the blood stream of critically ill and out-of-hospital cardiac arrest patients [27,28]. Aside from that, the deposition of NETs is associated with thromboinflammation and increased thrombus firmness [29]. Neutrophils have also been attributed a role in the initiation of thrombus formation in an intense cross-talk with platelets at the site of an injury [30]. Furthermore, initial thrombin generation during blood clotting has been attributed to them [31]. In summary, these characteristics might underline the essential role of neutrophils at the cross-roads between inflammation, hemostasis, and tissue remodeling. However, they do not appear to be associated with acute, premature clot breakdown in OHCA patients. This was also shown by the presence of higher levels of d-dimers in HF patients, which suggests specific enzymatic fibrin cleavage occurs rather than generalized matrix degradation by proteases. Fibrinolytic activation is common in severely injured and out-of-hospital cardiac arrest patients and is associated with a poor outcome [2,9]. Fibrinolysis, as a part of cell-based hemostasis is crucial to maintain blood flow and eventually represents the interface to subsequent wound-healing and regeneration. In cardiac arrest, the activation of pathways that maintain blood fluidity may represent a last resort in maintaining perfusion of vital organs. However, the cellular and plasmatic key factors and inhibitors involved in this process remain poorly understood. Reperfusion after tourniquet-induced ischemia results in elevated activated neutrophil counts, followed by a peak in fibrin degradation products [32]. Therefore, neutrophil fibrinolytic activity by live cells and in the shape of NET-complexed elastase might be another common denominator of the hypoperfusion of trauma and OHCA. However, we do not observe elevation of either histonylated DNA fragments or neutrophil elastase in patients with HF when compared to non-HF. Similarly, interleukin-6 (IL-6), an early pro-inflammatory cytokine known for its predictive value for massive transfusion requirement and, after successful resuscitation, is not elevated in hyperfibrinolytic patients [33,34]. However, cytokine levels such as IL-6 vary greatly depending on the timing of blood withdrawal and the underlying condition that leads to cardiac arrest. In an attempt to investigate inflammatory activation as a contributing factor to HF in OHCA, we performed the described assays on plasma samples in addition to the analyses of known regulators of fibrinolysis. With regard to these classic mediators, we do not find altered antigen concentrations of the classic fibrinolytic regulators tissue plasminogen activator (t-PA) and plasminogen activator inhibitor type-1 (PAI-1) in HF patients. The canonical fibrinolytic pathway is largely initiated by the activated and hypoxic vasculature through the release of t-PA from endothelial cells. T-PA activates plasminogen together with fibrin and enables its degradation by plasmin. PAI-1 serves as a primary inhibitor of this process by complexing t-PA and rendering it inactive. We find t-PA activity to be higher in HF patients, which is paralleled by a lower level of t-PA–PAI-1 complex in these patients. All OHCA patients show a similar magnitude of thrombin formation, as indicated by equal levels of TAT in both the HF and non-HF group. STM, a marker of endothelial cell damage and an indicator for the activation of the PC pathway, is equal in both groups. Higher t-PA activity in HF patients is paralleled by smaller amounts of t-PA–PAI-1 complex, higher levels of plasmin–antiplasmin, and, finally, fibrin degradation metabolites (d-dimer). APC is a known driver of HF in trauma and represents a potential interface to relevant inflammatory mechanisms. In the current study, the cytoprotective role of APC in the prevention of NETosis appears secondary in cardiac arrest. Although unrelated to HF, cardiac arrest appears to result in circulating histonylated DNA fragments and neutrophil elastase, both of which are features of neutrophil trap formation, but also signs of generalized cellular decay. 

## 5. Limitations

Given that this study is a follow-up of the 2013 hyperfibrinolysis in cardiac arrest (HICA) study, we were limited by the number of remaining patient samples available for immunoassays (41 patients out of 53). As a matter of fact, the small sample size also limited our ability to investigate the predictive value of the analyzed mediators for the return of spontaneous circulation (ROSC) and overall outcome. However, the association of hyperfibrinolysis with outcome has been shown in larger cohorts by other groups [35,36]. In addition to the exTEM^®^ assay that was performed in the HICA study, thromboelastometry in the presence of fibrinolysis inhibitors (e.g., apTEM^®^, TEM Innovations, Munich, Germany) could further substantiate our findings. We are also aware that in order to draw a detailed picture of the APC pathway in hypoperfusion, anticoagulation, and cell death, numerous other mediators (including single coagulation factor activity) need to be studied. Finally, the demonstrated association between isolated mediators in plasma and increased fibrinolytic activity in HF does not prove a cause–effect relationship. Limited to stable, circulating antigens in plasma, we referred to APC–PCI, TAT, and PAP as surrogate markers for the activation of protein C, thrombin formation, and plasmin activation. At the same time, the detection of higher levels of complexed mediators might inversely suggest a higher level of inhibition and thereby indicate diminished function. Apart from that, there is a lack of experimental evidence on the stability of the investigated analytes in plasma at −80 °C. We hope that future studies will pick up on the matter and help elucidate further the involved mechanisms and the potential implications for clinical decision-making. 

## 6. Conclusions

We confirm that hypoperfusion, the activation of protein C, and classic fibrinolytic pathways orchestrate HF in cardiac arrest. Just as in trauma-associated HF, the HF of OHCA is accompanied by an excessive release of t-PA and a more pronounced degradation of fibrin by plasmin. In general, HF, therefore, appears to be primarily driven by hypoxia and conventional pathways of fibrinolysis. Although early inflammatory activation, cell death, and features of neutrophil extracellular trap formation are present in out-of-hospital cardiac arrest, they appear unrelated to HF. Neutrophil elastases play a role in fibrin degradation during tissue remodeling, but do not appear to be confounders of acute premature fibrin clot breakdown in blood coagulation.

## Figures and Tables

**Table 1:** Patient characteristics and assessed parameters in plasma.

	ALL	non-HF	HF	*p*-Value	Summary
Age (years)	66 (58–71.5)	64 (53.5–71.5)	67 (60–72)	0.6406	ns
ROSC (yes/no)	19/22	13/12	10/6	0.5224	ns
Bystander CPR (yes/no)	29/12	19/6	10/6	0.485	ns
sTM (ng/mL)	38.13 (30.32–47.98)	36.55 (31.09–45.04)	41.49 (29.01–63.04)	0.4079	ns
APC–PCI (ng/mL)	9.96 (6.4–43.35)	8.33 (5.36–15.42)	15.83 (8.553–72.7)	0.0367	*
TAT (uG/mL)	122.8 (50.45–261.7)	76.1 (38.8–225.2)	180.7 (95.28–297)	0.0837	ns
D-dimer (mg/L)	7.7 (1.4–23.95)	2.4 (0.65–17)	14.9 (6.65–59.55)	0.014	*
PAP (mg/mL)	16,746 (1349–53,232)	8057 (546–19,099)	39,790 (22347–118,289)	0.0003	***
IL-6 (pg/mL)	18.7 (11.55–37.3)	18.7 (9.95–33.15)	18.55 (14.05–44.59)	0.4872	ns
t-PA activity (U/mL)	8.532 (0.3005–18.09)	4.82 (0.1662–8.712)	16.86 (12.44–30.45)	0.0005	***
t-PA antigen (ng/mL)	78.3 (54.88–98.28)	77.41 (49.01–94.96)	85.73 (62.41–119.1)	0.3073	ns
PAI-1 antigen (ng/mL)	173.1 (90.59–325.8)	168 (91.73–225.1)	203.4 (58.62–348)	0.7662	ns
t-PA–PAI-1 complex (ng/mL)	54.47 (34.54–96.91)	67.82 (39.5–129.9)	41.12 (30.88–55.07)	0.0373	*
PMN Elastase (ng/mL)	178.5 (115.5–347.5)	170 (104.6–320.4)	230.9 (126.9–455.3)	0.2593	ns
hcDNA (OD)	1.158 (0.413–2.018)	1.016 (0.3464–1.67)	1.466 (0.7427–2.272)	0.2268	ns

Data are given as patient numbers or median and interquartile range. ROSC: return of spontaneous circulation; CPR: cardiopulmonary resuscitation; sTM: soluble thrombomodulin; APC–PCI: activated protein C–protein C inhibitor complex; TAT: thrombin–antithrombin; PAP: plasmin–antiplasmin; IL-6: interleukin-6; t-PA: tissue plasminogen activator; PAI-1: plasminogen activator inhibitor-1; PMN: polymorphonuclear neutrophil; hcDNA: histonylated–citrullinated deoxyribonucleic acid; OD: optical density; HF: hyperfibrinolysis; non-HF: maximum lysis < 15%; HF: maximum lysis > 15%; ns: non-significant. *: *p* < 0.05; ***: *p* < 0.001.

## Data Availability

The data presented in this study are available on request from the corresponding author.

## Abbreviations

APC	Activated protein C
ELISA	Enzyme-linked immuno-sorbent assay
EPCR	Endothelial protein C receptor
hDNA	Histonylated deoxyribonucleic acid
HF	Hyperfibrinolysis
PAI-1	Plasminogen activator inhibitor 1
PCI	Protein C inhibitor
PMA	Phorbol 12-myristate 13-acetate
PMN	Polymorphonuclear neutrophil
MCF	Maximum clot firmness
ML	Maximum lysis
NET	Neutrophil extracellular traps
OHCA	Out-of-hospital cardiac arrest
ROTEM	Rotational thromboelastometry
sTM	Soluble thrombomodulin
TIC	Trauma-induced coagulopathy
t-PA	Tissue plasminogen activator

## References

[1] Brohi K., Cohen M.J., Ganter M.T., Schultz M.J., Levi M., Mackersie R.C., Pittet J.F. (2008). Acute coagulopathy of trauma: Hypoperfusion induces systemic anticoagulation and hyperfibrinolysis. J. Trauma.

[2] Schöchl H., Cadamuro J., Seidl S., Franz A., Solomon C., Schlimp C.J., Ziegler B. (2013). Hyperfibrinolysis is common in out-of-hospital cardiac arrest: Results from a prospective observational thromboelastometry study. Resuscitation.

[3] Brohi K., Cohen M.J., Ganter M.T., Matthay M.A., Mackersie R.C., Pittet J.F. (2007). Acute traumatic coagulopathy: Initiated by hypo-perfusion: Modulated through the protein C pathway?. Ann. Surg..

[4] Chang R., Cardenas J.C., Wade C.E., Holcomb J.B. (2016). Advances in the understanding of trauma-induced coagulopathy. Blood.

[5] Davenport R.A., Brohi K. (2016). Cause of trauma-induced coagulopathy. Curr. Opin. Anaesthesiol..

[6] Hunt B.J., Jurd K.M. (1998). Endothelial cell activation. Bmj.

[7] Johansson P.I., Sørensen A.M., Perner A., Welling K.L., Wanscher M., Larsen C.F., Ostrowski S.R. (2011). Disseminated intravascular coagulation or acute coagulopathy of trauma shock early after trauma? An observational study. Crit. Care.

[8] Moore E.E., Moore H.B., Kornblith L.Z., Neal M.D., Hoffman M., Mutch N.J., Schöchl H., Hunt B.J., Sauaia A. (2021). Trauma-induced coagulopathy. Nat. Rev. Dis. Prim..

[9] Schöchl H., Frietsch T., Pavelka M., Jámbor C. (2009). Hyperfibrinolysis After Major Trauma: Differential Diagnosis of Lysis Patterns and Prognostic Value of Thrombelastometry. J. Trauma Inj. Infect. Crit. Care.

[10] Huber D., Cramer E.M., Kaufmann J.E., Meda P., Massé J.-M., Kruithof E.K.O., Vischer U.M. (2002). Tissue-type plasminogen activator (t-PA) is stored in Weibel-Palade bodies in human endothelial cells both in vitro and in vivo. Blood.

[11] Cap A., Hunt B.J. (2015). The pathogenesis of traumatic coagulopathy. Anaesthesia.

[12] Healy L.D., Rigg R.A., Griffin J.H., McCarty O.J.T. (2018). Regulation of immune cell signaling by activated protein C. J. Leukoc. Biol..

[13] Davenport R.A., Guerreiro M.M., Frith D., Rourke B.C., Platton B.S., Cohen M.M., Pearse R., Thiemermann C., Brohi M.K. (2017). Activated Protein C Drives the Hyperfibrinolysis of Acute Traumatic Coagulopathy. Anesthesiology.

[14] Iba T., Nagakari K. (2015). The effect of plasma-derived activated protein C on leukocyte cell-death and vascular endothelial damage. Thromb. Res..

[15] Brinkmann V., Reichard U., Goosmann C., Fauler B., Uhlemann Y., Weiss D.S., Weinrauch Y., Zychlinsky A. (2004). Neutrophil extracellular traps kill bacteria. Science.

[16] Mukhopadhyay S., Johnson T.A., Duru N., Buzza M.S., Pawar N.R., Sarkar R., Antalis T.M. (2019). Fibrinolysis and Inflammation in Venous Thrombus Resolution. Front. Immunol..

[17] Schwameis M., Schober A., Schoergenhofer C., Sperr W.R., Schöchl H., Janata-Schwatczek K., Kürkciyan E.I., Sterz F., Jilma B. (2015). Asphyxia by Drowning Induces Massive Bleeding Due to Hyperfibrinolytic Disseminated Intravascular Coagulation. Crit. Care Med..

[18] de Oliveira T.H.C., Marques P.E., Proost P., Teixeira M.M.M. (2018). Neutrophils: A cornerstone of liver ischemia and reperfusion injury. Lab. Investig..

[19] Adams S.A., Kelly S.L., Kirsch R.E., Robson S.C., Shephard E.G. (1995). Role of neutrophil membrane proteases in fibrin degradation. Blood Coagul. Fibrinolysis.

[20] Johansson P., Stensballe J., Ostrowski S. (2017). Shock induced endotheliopathy (SHINE) in acute critical illness—A unifying pathophysiologic mechanism. Crit. Care.

[21] Hofmann N., Zipperle J., Brettner F., Jafarmadar M., Ashmwe M., Keibl C., Ponschab M., Kipman U., Bahrami A., Redl H. (2019). Effect of Coagulation Factor Concentrates on Markers of Endothelial Cell Damage in Experimental Hemorrhagic Shock. Shock.

[22] Viersen V., Greuters S., Korfage A., Van der Rijst C., Van Bochove V., Nanayakkara P., Vandewalle E., Boer C. (2012). Hyperfibrinolysis in out of hospital cardiac arrest is associated with markers of hypoperfusion. Resuscitation.

[23] Menges T., Engel J., Welters I., Wagner R.M., Little S., Ruwoldt R., Wollbrueck M., Hempelmann G. (1999). Changes in blood lymphocyte populations after multiple trauma: Association with post-traumatic complications. Crit. Care Med..

[24] Walsh D.S., Siritongtaworn P., Pattanapanyasat K., Thavichaigarn P., Kongcharoen P., Jiarakul N., Tongtawe P., Yongvanitchit K., Komoltri C., Dheeradhada C. (2000). Lymphocyte activation after nonthermal trauma. Br. J. Surg..

[25] Machovic R., Himer A., Owen W.G. (1990). Neutrophil proteases in plasminogen activation. Blood Coag Fibrinolysis.

[26] Hirose T., Hamaguchi S., Matsumoto N., Irisawa T., Seki M., Tasaki O., Hosotsubo H., Yamamoto N., Yamamoto K., Akeda Y. (2014). Presence of Neutrophil Extracellular Traps and Citrullinated Histone H3 in the Bloodstream of Critically Ill Patients. PLoS ONE.

[27] Johansson P., Windeløv N., Rasmussen L.S., Sørensen A., Ostrowski S.R. (2013). Blood levels of histone-complexed DNA fragments are associated with coagulopathy, inflammation and endothelial damage early after trauma. J. Emergencies Trauma Shock.

[28] Mauracher L.M., Buchtele N., Schörgenhofer C., Weiser C., Herkner H., Merrelaar A., Spiel A.O., Hell L., Ay C., Pabinger I. (2019). Increased Citrullinated Histone H3 Levels in the Early Post-Resuscitative Period Are Associated with Poor Neurologic Function in Cardiac Arrest Survivors-A Prospective Observational Study. J. Clin. Med..

[29] Mangold A., Alias S., Scherz T., Hofbauer T., Jakowitsch J., Panzenböck A., Simon D., Laimer D., Bangert C., Kammerlander A. (2015). Coronary neutrophil extracellular trap burden and deoxyribonuclease activity in ST-elevation acute coronary syndrome are predictors of ST-segment resolution and infarct size. Circ. Res..

[30] Massberg S., Grahl L., von Bruehl M.L., Manukyan D., Pfeiler S., Goosmann C., Brinkmann V., Lorenz M., Bidzhekov K., Khandagale A.B. (2010). Reciprocal coupling of coagulation and innate immunity via neutrophil serine proteases. Nat. Med..

[31] Darbousset R., Delierneux C., Mezouar S., Hego A., Lecut C., Guillaumat I., Riederer M.A., Evans R.J., Dignat-George F., Panicot-Dubois L. (2014). P2X1 expressed on polymorphonuclear neutrophils and platelets is required for thrombosis in mice. Blood.

[32] Hughes S.F., Hendricks B.D., Edwards D.R., Bastawrous S.S., Roberts G.E., Middleton J.F. (2007). Mild episodes of tourniquet-induced forearm ischaemia-reperfusion injury results in leukocyte activation and changes in inflammatory and coagulation markers. J. Inflamm..

[33] Bro-Jeppesen J., Kjaergaard J., Stammet P., Wise M.P., Hovdenes J., Åneman A., Horn J., Devaux Y., Erlinge D., Gasche Y. (2016). TTM-Trial Investigators. Predictive value of interleukin-6 in post-cardiac arrest patients treated with targeted temperature management at 33 °C or 36 °C. Resuscitation.

[34] Weichselbaum N., Oberladstätter D., Schlimp C.J., Zipperle J., Voelckel W., Grottke O., Zimmermann G., Osuchowski M., Schöchl H. (2021). High Interleukin-6 Plasma Concentration upon Admission Is Predictive of Massive Transfusion in Severely Injured Patients. J. Clin. Med..

[35] Wada T., Gando S., Ono Y., Maekawa K., Katabami K., Hayakawa M., Sawamura A. (2016). Disseminated intravascular coagulation with the fibrinolytic phenotype predicts the outcome of patients with out-of-hospital cardiac arrest. Thromb. J..

[36] Buchtele N., Schörgenhofer C., Spiel A.O., Jilma B., Schwameis M. (2018). Increased Fibrinolysis as a Specific Marker of Poor Outcome After Cardiac Arrest. Crit. Care Med..

